# An AI-driven multiscale methodology to develop transparent wood as sustainable functional material by using the SSbD concept

**DOI:** 10.1016/j.csbj.2024.10.022

**Published:** 2024-10-18

**Authors:** Päivi Kivikytö-Reponen, Stefania Fortino, Veera Marttila, Alexey Khakalo, Kari Kolari, Antti Puisto, Daniele Nuvoli, Alberto Mariani

**Affiliations:** a VTT Technical Research Centre of Finland, VTT, PL 1000, 02044, Finland; b Università degli Studi di Sassari, Dipartimento di Scienze Chimiche, Fisiche, Matematiche e Naturali, Via Vienna, 2, 07100 Sassari, Italy

**Keywords:** Transparent wood, AI-driven, Multiscale modeling, Functional material, Safe and sustainable by design, SSbD

## Abstract

Efficient design, production, and optimization of new safe and sustainable by design materials for various industrial sectors is an on-going challenge for our society, poised to escalate in the future. Wood-based composite materials offer an attractive sustainable alternative to high impact materials such as glass and polymers and have been the focus of experimental research and development for years. Computational and AI-based materials design provides significant speed-up the development of these materials compared to traditional methods of development. However, reliable numerical models are essential for achieving this goal. The AI-TranspWood project, recently funded by the European Commission, has the ambition to develop such computational and AI-based tools in the context of transparent wood (TW), a promising composite with potential applications in various industrial fields. In this project we advance the development specifically by using an Artificial Intelligence (AI)-driven multiscale methodology.

## Graphical Abstract

**Figure:**
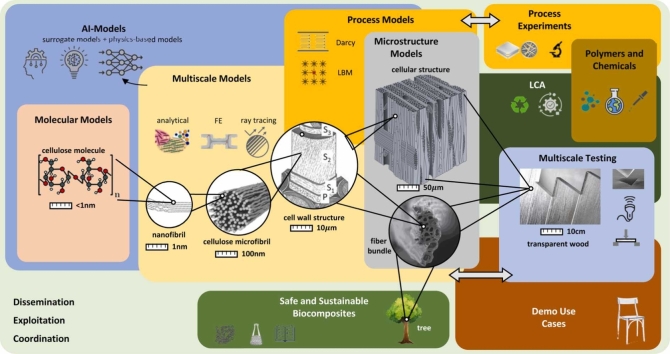

## 1 Introduction

Efficient design, production, and optimization of new safe and sustainable by design materials for various industrial sectors is an on-going challenge for our society, poised to escalate in the future. Wood-based composites offer an attractive sustainable alternative to high impact materials such as glass and polymers and have been the focus of experimental research and development for years. Computational and Artificial Intelligence (AI)-based materials design provides significant speed-up the development of these materials compared to traditional methods. However, reliable numerical models are essential for achieving this goal. The AI-TranspWood project recently funded by the European Commission, has the ambition to develop such computational and AI-based tools in the context of transparent wood (TW), a promising composite with potential applications in various industrial fields. The project is coordinated by VTT Technical Research Centre of Finland Ltd (Finland) and composed of thirteen partners. In addition to VTT, the RTO partners organisations are: Aalto University (Finland), KTH Royal Institute of Technology (Sweden), University of Sassari (Italy), Politecnico di Torino (Italy), Technical University of Vienna (Austria), AIMPLAS Plastics Technology Centre (Spain). The industrial partners are: Latvijas Finieris (Latvia), Oyak Renault (Turkey), Inuru (Germany), BM Plastic (Italy), and Strane Innovation (France). In addition, we have an associate partner, the Ecole Polytechnique Federale de Lausanne, Centre Européen de Calcul Atomique et Moléculaire (Switzerland). In this project we advance the TW development specifically by using an AI-driven multiscale methodology. More details on the consortium can be found in the project website (https://www.ai-transpwood-project.eu/) [1].

### 1.1 Transparent wood

Transparent wood acquired attention since its discovery and morphological characterization in 1992 by S. Fink [2]. Its complex microstructure originates from that of pristine wood, and the resulting composite is heterogeneous across the scales, providing interesting features: it is lightweight, transparent to visible light, and, by subsequent treatments (i.e., modification, infiltration with suitable resins and possibly functionalization) it becomes the best candidate for replacing glass and some plastics in a variety of applications.

In the last decade, there were important advances in the research and development on delignification and functionalization of wood to create new materials such as TW [3], [4], [5], [6]. However, the European market for TW composites is still new, and a recent report [7] estimates that the global Transparent Wood industry will generate $208.1 million by 2031 witnessing a Compound Annual Growth Rate (CAGR) of 9.0 % from 2022 to 2031, due to TW emerging as a promising alternative to petroleum-based harmful plastics [7]. Being more sustainable, lighter, stronger, and having a lower carbon footprint compared to traditional building materials, it is expected to become a popular choice for energy-efficient buildings and sustainable construction projects.

Typically, the production of transparent wood includes several chemical modification methods such as delignification, and monomer infiltration. Delignification is performed to partly remove light absorbing lignin and to increase the porosity of the wood. In the following step, delignified wood is infiltrated with a monomer the refractive index of which, after polymerization, matches that of cellulose and hemicellulose remaining in the delignified structures.

## 2 Project description

The project *‘AI-driven multiscale methodology to develop Transparent Wood as sustainable functional material’* (AI-TranspWood), funded by the European Commission within the call HORIZON-CL4–2023-RESILIENCE-01–23, aims to create an AI-driven multiscale methodology within the Safe and Sustainable by Design (SSbD) framework for functional wood-based composites. The concept will be demonstrated for Transparent Wood (TW), a promising material with potential applications in several industrial fields, such as construction, automotive, electronics, and furniture. Utilizing AI tools and conducting advanced experiments, we aim to create multiscale models spanning from the atomistic to continuum scales. These models will address the manufacturing and mechanical aspects of transparent wood and facilitate virtual screening of bio-sourced alternatives for the chemicals used in TW production.

The AI-TranspWood project integrates efficiently advanced AI-driven computational models with the SSbD framework for wood-based composites and demonstrates the methodology for TW, with the vision to substitute substances of concern such as petrochemical-based plastics and glass in key applications. This will be possible thanks to the user-oriented design tools made available for industrial users.

### 2.1 AI-driven multiscale methodology

Computational modelling of TW properties requires specific physics-based modelling approaches spanning across several scales and physical domains. Such approaches are restricted by the computational power available, and the profound knowledge required. At the same time, developing procedures for producing TW, and required laboratory experiments are needed to give input data for modelling and on the other hand for validation of the models.

**Fig. 1:**
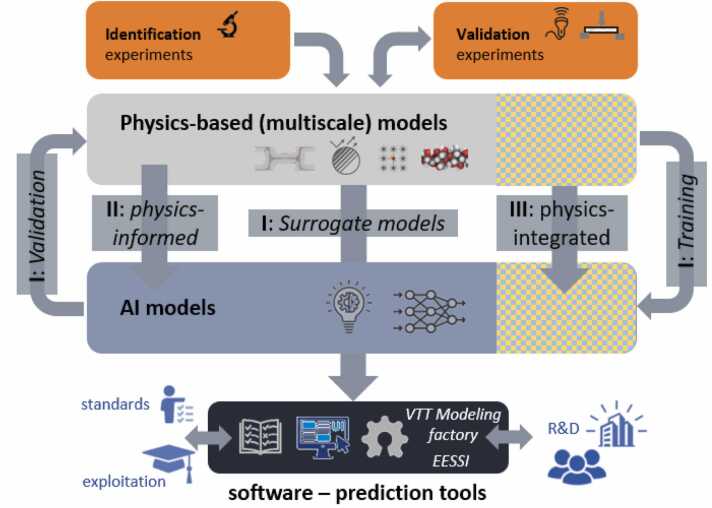
Conceptual three-stage process to link physics-based models to AI-methodology, and model classes which will then be implemented in predictive software.

AI-TranspWood proposes a systematic approach for complementing physics-based modelling and providing tractable software tools with the help of AI and machine learning (ML), as schematized in Fig. 1. It leverages physics-based models to develop AI-driven surrogate models aimed at accelerating the simulation workflows, and to make them accessible to users without the necessary high-performance computing capabilities. These surrogate models are then further improved by integrating physics sub-models directly into the AI methodology. Finally, advanced physics models, such as non-linear finite element approaches, are linked to AI. The entire reality described by physics models is then connected to AI technology handling aspects not accommodated by physics-based models, e.g., geometric variability and material uncertainties due to growth and production irregularities.

The developed models built will be freely available in EESSI environment [8] for researchers and other end users. Selected demo cases along with LCA tools are made available for industrial users through the VTT Modeling Factory [9].

## 3 Impact, project pre-results

Among the first results of AI-TranspWood project, a general procedure for producing TW was developed. This involved the identification of SSbD requirements and key performance indicators. In particular, the first activities were aimed at connecting the SSbD concept to the design process and the generation of the safe and sustainable by design principles, which was conducted by a collaboration among experimental, computational and industrial partners.

### 3.1 Procedure of TW production

The general procedure for producing TW from balsa or birch wood, with hydroxyethyl methacrylate (HEMA, an easily polymerizable chemical used to make soft, flexible materials like contact lenses and dental products), was adopted at University of Sassari in the AI-TranspWood project and is described in the following.

**Fig. 2:**
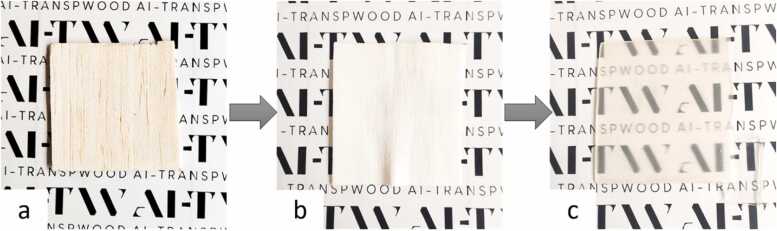
Images of balsa wood at various stages of the transparent wood production process. Balsa wood (left), delignified balsa wood (centre), and transparent balsa wood with p(HEMA) (right).

Source: University of Sassari.

Balsa wood (1 mm thick) or birch wood (1.5 mm thick) is cut into 5 cm squares. Complete delignification is achieved by immersing the wood samples in a buffer solution at pH 4.6 containing 1 wt% sodium chlorite, at a temperature of 80 °C for a total time of 18 h. The delignified wood is then washed with water, ethanol, and finally acetone. Infiltration is carried out under vacuum for a total time of 18 h, immersing the delignified wood in the monomer (HEMA) containing 0.1 mol% benzoyl peroxide (thermal initiator). The infiltrated wood is then sealed between two glass plates and placed in an oven at 80 °C for 4 h to allow complete polymerization of HEMA. An example of 5 cm square original balsa wood, delignified wood and TW sample is shown in Fig. 2.

The materials and their quantities used to produce the TW samples are summarized in Table 1. In a successive step of the project, larger samples (up to 23×23 cm^2^) will be produced with the aim to build demonstrators in focused industrial fields.

**Table 1:** Materials and quantities used to obtain TW samples with dimensions 5×5 cm^2^.

**Material**	**Quantity**
Balsa wood	0.18 g
Birch wood	2.4 g
Sodium chlorite solution	150 ml
Water	90 ml
Ethanol	90 ml
Acetone	90 ml
HEMA	20 ml (21.4 g)
Benzoyl peroxide	0.04 g

The information about materials produced in AI-TranspWood will be used within the SSbD concept illustrated in the next section.

### 3.2 Safe and sustainable by design concept

The work about Safe and sustainable by design (SSbD) is a task specifically lead by VTT within AI-TranspWood. The SSbD is a design approach where objectives such as minimizing hazardous chemicals use, reducing greenhouse gas emissions [10], and fostering materials reuse and recycling are built into product design.

The SSbD is a voluntary framework that can be applied in the innovation or (re)design of chemicals and materials. The framework development is still progressing and the current European Commission recommendation [11] and the Joint Research Centre (JRC) guidance reports [11] are the main and most detailed instructions for the implementation of the approach. The framework consists of a (re)design phase and a 4-step assessment phase. The current guidance heavily focuses on assessments to support the safe and sustainable design of a material or chemical. The assessments consist of hazard, safety, and environmental aspects, applying, among others, the life cycle assessment method. On the other hand, European Chemical Industry Council (Cefic) has produced instructions for the chemical industry on the SSbD framework [12]. In the Cefic approach, the connection to research and development is highlighted by dedicating the first three activities (out of five in total) to determining design characteristics and the final two activities to assessments and trade-off analysis. During the first steps the performance and functionality needs are defined, and the creation of minimum requirements and dimensions of improvement are identified. Based on these factors, the final design principles are chosen to support the safe and sustainable design.

**Fig. 3:**
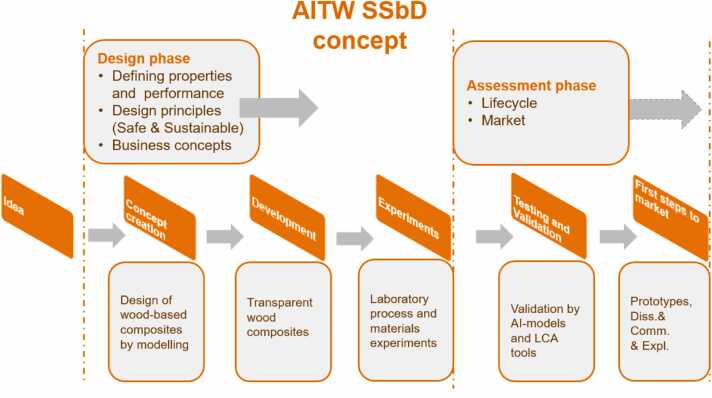
Scheme of the SSbD concept in AI-TranspWood project.

In the AI-TranspWood project, a formulation of the SSbD concept is defined for the purpose of screening polymers and chemicals selected for the TW material and the final composites. This is accomplished by using the data generated by the models and assessments (e.g. safety, hazard, LCA, and LCC), identifying their safety and environmental and economic hot spots. A scheme of the SSbD is presented in Fig. 3^.^ The SSbD approach is an iterative process, where the feedback from the screening study helps the material developers to improve the environmental performance at an early stage.

The SSbD development within AI-TranspWood followed the activities proposed by Cefic [12].

At the beginning of the project, the design phase work started with a focused workshop between experimental and computational project partners. [1] Cefic’s approach proposes guiding design principles or dimensions to be assessed at the level of product-application combination in a stage-gate-like approach during innovation. However, the specific performance requirements for different applications such as lighting and decorative applications will be addressed later in the project. As results of the workshop, the general (not application specific) requirements and key performance indicators (KPIs) for the performance of TW were summarized in Table 2, and safety and sustainability requirements and KPIs (Activity 1) for TW manufacturing were listed in Table 3. TW is a material suitable for many applications, and the final performance requirements (e.g. mechanical strength, transparency) are mainly determined by the application and its related KPIs. These Table 2, Table 3 will be updated during the project. For instance, the final Table 2 will include information such as the ones provided in Table 1.

**Table 2:** Design principles for the performance of TW, collected from AI-TranspWood partners in project workshop.

**Themes**	**Requirements**	**KPIs**
Performance	Raw woods should have suitable mechanical properties.	Mechanical strength
	Raw woods should have suitable channels to make delignification and infiltration efficient and fast.	Amount of removed lignin, amount of monomer infiltrated.
	Monomers should have affinity toward the internal functional groups of wood, and their viscosity should be low enough to facilitate their infiltration.	Amount of monomer entered the wood channels.
	Monomers should have low volatility and be polymerized in mild conditions avoiding any bubbling.	Transparency
	Polymers should possess good mechanical properties	Mechanical properties of the transparent wood.
	Polymers must be amorphous and have a refractive index matching that of the delignified wood.	Transparency
	The process should not produce wastes (co- or by-products)	Polymer yield; amount of co- and by-products.
	The process should be easily scalable.	Process scalability

**Table 3:** SSbD themes, requirements, and KPIs, collected from AI-TranspWood partners.

**Themes**	**Requirements**	**KPIs**
Safety	The chemicals and materials must comply with REACH, with no toxicity, no microplastics, and minimal chemical exposure.	Compliance Toxicity Microplastics Chemical exposure
Environmental impacts	The monomers must be biobased if possible. The availability of biobased raw materials can be a challenge.	Biobased content %
	The manufacturing should not produce any pollution or residue.	Emissions to air, water and soil Waste
	With better End-of-Life assessments and increasing the recyclability of transparent wood environmental impacts could be mitigated and the circularity of the materials enhanced.	Resources and waste
	Minimizing need for electricity and maximizing green energy,	Energy efficiency, energy use Energy mix
	Forestry and trees	Biodiversity and ecosystem impacts
Social impacts	The final products must be accepted by the citizens and industry and can also create social impact by increasing job positions in the industry.	
	It must be compliant with standards and legislation and cannot cause any health risks.	Compliance with legislation
Economic	The product and its production must be cost-effective and profitable. E.g. the availability of raw materials can induce costs. The trade-offs between economic and other requirements/key performance indicators should be considered.	Life cycle cost
	The market potential was also emphasized. Scenarios on future demand, identification of competitors, and the current market situation should be taken into account.	Market potential

### 3.3 Material modelling

Safety and minimizing environmental impact are central objectives of the Safe and Sustainable by Design framework. Material modelling contributes to this goal by enabling the estimation of resource consumption during manufacturing. Referring to Fig. 1, in the AI-TranspWood project, this is achieved by resolving the relationship between the atomistic structure of materials and their rheological behaviour with the help of AI. Rheological models can then be integrated into process simulators to estimate the energy required for material processing. Both the energy estimates and the material's chemical composition can be incorporated into the SSbD framework to assess environmental impacts.

Equally important within the SSbD context is determining how long a material remains functional in its operational environment. By understanding the material's degradation mechanisms under various environmental conditions, it becomes possible to simulate its degradation over time. Establishing criteria for acceptable tolerances for the key functionalities allows for the determination of the material’s usable lifespan.

## 4 Discussion

The SSbD activities within the AI-TranspWood project identified several key focus dimensions of TW’s design process to meet EU’s Green Deal goals. These include prioritizing the use of renewable and biobased sources, compliance with the current legislation with avoiding hazardous emissions, minimising the use of hazardous chemicals, mitigating climate change over the whole life cycle, and designing for end-of-life to increase circularity. As SSbD is a tiered and iterative process, the minimum requirements and dimensions of improvement will be formed later to support the design based on three pillars of sustainability while obtaining compliance with regulation.

TW is a new material entering the market. Therefore, design principles are important to be formulated at this point for the material itself, and later for TW materials used for specific applications. Design principles for performance, safety, and sustainability targeting a specific application depend on the end-use application. However, preliminary design principles for the lifecycle of TW composites can be generated already at this early stage. Later in the development process, during the product design phase, the design principles are reviewed and fixed to support a specific product performance.

### 4.1 Preliminary design principles for the lifecycle of TW composites

Based on the knowledge gathered from a focused project workshop, focused design principles were determined. The formulation of preliminary design principles for TW and its raw materials are the following:

• TW design principles support optimal performance that is defined by final mechanical and functional (mainly, optical) properties in AI-TranspWood project. These properties are dependent on the final application. For example, from a sustainability point of view (energy efficiency), thermal conductivity is relevant.
• Raw material requirements in general for TW: lifecycle design and preferences on biobased raw materials if performance, safety, and sustainability requirements are fulfilled. For example, the applied monomers are biobased when possible. As new materials are developed, the sustainable process development is done at the same time, including end-of-life recycling processes. The processes should be scalable and with high yield. The manufacturing and recycling process emissions, by-products, and waste materials should be minimized.
• Safety issues, especially toxicity, and chemical exposure are in focus, compliance with regulation and beyond.
• Environmental impacts of TW are minimized by designing the whole lifecycle until the end of life, minimizing the materials, energy, emissions, and residues through the TW lifecycle.
• Final products need to have compliance with regulations and standards and meet the acceptance of the users. The raw materials applied in TW manufacturing are socially responsible produced.
• Economic value of raw materials, processes, especially from a lifecycle perspective, are optimized.

### 4.2 SSbD reflection to Cefic activity 3 and JRC guidelines

Both the European Chemical Industry Council (Cefic) [12] and the JRC [11] give guidelines and examples for formulation of design principles. In Cefic’s guidance, design principles are covered by Activity 3, and they are advised to be chosen based on dimensions and the chosen use case. These principles can target to set criteria, for example from risk assessment and management, environmental impact categories perspective or through other focus dimensions outlined in the Cefic approach. On the other hand, JRC guideline mentions design principles related to actions and expands to the whole lifecycle in their guidelines, presenting design principles SSbD1–8 such as materials efficiency, minimal use of hazardous materials, energy efficiency, use of renewable sources, avoidance of hazardous emissions, and exposure to hazardous substances, end-of-life design and whole lifecycle implementation.

The exposure and safety aspects of the substances applied can be addressed with risk assessment towards human health, implementing design principles such as monitoring adverse effects such as acute toxicity, eye irritations, or physical hazards, Risk assessments towards the environment could focus on principles such as ozone depleting substances or aquatic toxicities. On the other hand, JRC proposes a design principle can also be on a more general level such as the substitution of a substance of a very high concern (SVHC) with a safer alternative. Therefore, besides compliance with current safety regulation, the usage of SVHC is avoided.

## 5 Conclusions

The on-going experiments are essential in the first approach for supporting AI-driven multiscale methodology to design for lifecycle. Currently TW is on the development phase, and much focus is placed on the material development itself. At this stage, AI-TranspWood project follows the JRC strategy in design principles, although the process formulation followed Cefic activity steps. As a future work, the design principles are updated to support the final application design. There TW design is targeted to a specific application, among others, the lighting and interior design applications: electroluminescent lamps, OLEDs with integrated transparent wood materials for lighting, lighting guides, in mold decoration, sealing and ceiling decorative panels, interior walls, furniture components, and plywood.

After screening the components of transparent wood (i.e. monomers, chemicals, wood), SSbD implementation continues towards assessment of environmental impacts. This supports understanding of the sustainability of the whole service life of the selected materials and new TW products to be developed. The hazard and safety assessment work to support safe design will be done parallel to the environmental assessment. Finally, SSbD guidelines support the safe and sustainable design of use cases. Environmental impacts are evaluated with data and knowledge gained during the project. The LCA and environmental footprint methodology will consider the whole value chain of the production system to quantify environmental impacts. Also, circularity and R-strategies of selected polymers and chemicals, and biocomposites will be evaluated. For supporting the lifecycle of the products, the recycling of TW products will also be studied. Due to environmental and economic issues, mechanical recycling will be prioritized. However, due to the composition and nature of the polymers, chemical recycling will be required.

The aim of the project is to develop advanced TW through SSbD concept using AI-driven multiscale methodology. The communication between LCA and the material surrogate models published on open platforms will be created. By developing AI-supported SSbD framework for TW, we contribute to the European Green Deal by providing innovative sustainable materials and cost-effective virtual design tools for European industries, paving the way towards green and sustainable transition. SSbD tools used by the chemicals and materials community with new transparent wood materials will increase the innovation capacity of SMEs and industry for future sustainable products.

## CRediT authorship contribution statement

**Stefania Fortino:** Writing – original draft, Project administration, Methodology, Funding acquisition, Conceptualization. **Veera Marttila:** Writing – original draft. **Päivi Kivikytö-Reponen:** Writing – original draft, Methodology, Conceptualization. **Alberto Mariani:** Writing – original draft. **Antti Puisto:** Writing – original draft. **Daniele Nuvoli:** Writing – original draft. **Alexey Khakalo:** Writing – original draft. **Kari Kolari:** Writing – original draft.

## Declaration of Competing Interest

We have no conflicts of interest to disclose.
